# Birth defects in Iraq and the plausibility of environmental exposure: A review

**DOI:** 10.1186/1752-1505-6-3

**Published:** 2012-07-28

**Authors:** Tariq S Al-Hadithi, Jawad K Al-Diwan, Abubakir M Saleh, Nazar P Shabila

**Affiliations:** 1 Department of Community Medicine, College of Medicine, Hawler Medical University, Erbil, Iraq; 2 Department of Community Medicine, College of Medicine, Baghdad University, Baghdad, Iraq

## Abstract

An increased prevalence of birth defects was allegedly reported in Iraq in the post 1991 Gulf War period, which was largely attributed to exposure to depleted uranium used in the war. This has encouraged further research on this particular topic. This paper reviews the published literature and provided evidence concerning birth defects in Iraq to elucidate possible environmental exposure. In addition to published research, this review used some direct observation of birth defects data from Al-Ramadi Maternity and Paediatric Hospital in Al-Anbar Governorate in Iraq from1^st^ July 2000 through 30^th^ June 2002. In addition to depleted uranium other war-related environmental factors have been studied and linked directly or indirectly with the increasing prevalence of birth defects. However, the reviewed studies and the available research evidence do not provide a clear increase in birth defects and a clear indication of a possible environmental exposure including depleted uranium although the country has been facing several environmental challenges since 1980.

## Introduction

Birth defects are still the leading cause of perinatal mortality and childhood disability in developed countries [1]. In contrast, in some developing countries where infant mortality remains very high, the leading causes of death are related to malnutrition and infection [2]. However, birth defects in the developing world are largely underreported by deficiencies in diagnostic capabilities and lack of reliability of medical records and health statistics [3]. Thus, an increase in birth defects rate should be handled with caution as this could only be attributed to use of more reliable diagnostic facilities or an improvement in medical records.

The causes of most birth defects remain unknown, but the growing literature indicates that environmental factors may cause genetic mutations and interact with genetic factors predisposing to birth defects [4]. Thus, most birth defects can be considered to be of multifactorial causation that is due to a combination of environmental and genetic factors. Prevalence studies of birth defects are useful to establish baseline rates, to document changes over time and to identify clues to etiology. They are also important for planning and evaluating antenatal screening for birth defects, particularly for high risk population [5].

Workers allegedly reported a high prevalence of birth defects in Basrah (south of Iraq) in the post 1991 Gulf War period, which was attributed to exposure to depleted uranium (DU) used in the war [6,7]. This provided impetus for further research in this particular field in Iraq. This paper reviews the published literature and provided evidence concerning birth defects in Iraq to elucidate possible environmental exposure. The review also used some directly collected data on birth defects from Al-Ramadi Maternity and Paediatric Hospital in Al-Anbar Governorate in Iraq from1^st^ July 2000 through 30^th^ June 2002 where a total of 12831 live births and stillbirths at hospital were included. All live born neonates were physically examined by a pediatrician during the first three days of delivery, and birth defects were reported. Stillborn fetuses were only physically examined without autopsy. This study is referred to as ‘Al-Anbar study’ in this review.

### Prevalence of birth defects in Iraq

Al-Anbar study revealed a rate of 8.5 birth defects per 1000 births. Similar rates were reported in Basrah in 1998 (7.76 per 1000 births) [6] and 1994 (8.7 per 1000 births) [8]. Recently a high rate of 12.36 per 1000 births was reported from Baghdad, Iraq in 2007 [9]. Most of these reported rates are generally lower than those reported in United Arab Emirates (10.5 per 1000 live births during 1992–1994) [10], Bahrain (18.75 per 1000 live births in 1985) [11], Turkey (11.1 per 1000 live births during 1988–1995) [12] and Iran (16.55 per 1000 total births which increased from 10.46 in 2000 to 17.01 per 1000 births in 2004) [13]. However, the rates of congenital defects reported in post-war period in Iraq and other Middle Eastern countries did not reach the western world levels (23.8 per 1000 live births in 1997 in Glasgow, UK [5], 19.5 per 1000 live births in the 16 European registries of EUROCAT for the period 1990–1994 [14] and approximately 30 per 1000 births in the United States) [15]. However, a study from Erbil, Iraq on live births between 1990 and 1999 revealed an unexpected high rate of 23.9 per 1000 live births, comparable to that of western countries [16]. This high rate could be attributed to the hospital-based reporting bias as the study was done on newborns admitted to neonate intensive care unit in addition to other methodological limitations.

### Patterns of birth defects

Central nervous system defects were the most common types of birth defects among live births. In Al-Anbar governorate it constituted 55% with a rate of 4.6 per 1000 live births. Similar rates were reported in Basrah for the period 1999–2000 (4.35 per 1000 births) [6], and Erbil (4.48 per 1000 live births [16]. Recently a similar rate of neural tube defects (NTDs) (4.7 per 1000 live births) was reported from Duhok, Iraq [17]. However, higher rates of NTDs were reported in Baghdad (5.95 per 1000 births) [9] and Diwaniyah (8.4 per 1000 total births in 2000) [18] of Iraq. Musculoskeletal malformations constituted 33% with a rate of 2.8 per 1000 live births while congenital health diseases constituted only 1.8% with a rate of 0.15 per 1000 live births. In Basrah studies for the period 1999–2000 the reported rates were 1.4 and 1.36 per 1000 births, respectively [6,7]. Gastrointestinal defects and genitourinary defects in Al-Anbar study accounted for 11.9% with a rate of 1.0 per 1000 live births and 1.8% with a rate of 0.15 per 1000 live births, respectively. In Iran study, anomalies of the nervous system, genitourinary system and limb anomalies accounted proportionally for more than 65% of anomalies [13]. In the Turkish study the most common anomalies were the central nervous system and urogenital anomalies (2.7 and 2.1 per 1000 births, respectively) [12]. In Bahrain anomalies of the musculoskeletal system had the highest frequency at an average of 2.8 per 1000 births and the incidence of NTDs was 1.5 per 1000 births [11].

### Causes of birth defects

The root causes of many adverse pregnancy outcomes are not well understood, but there is growing evidence that the environment can play an important role. ‘Environment” is a broad term that includes familiar contributors such as nutrition, adequacy of prenatal care, smoking and alcohol use, maternal age, and socioeconomic disparities, as well as less familiar contributors including pollution and chemical agents encountered both indoors and outdoors. In many cases, two or more environmental factors may be interrelated or synergistic [19].

Based on the current knowledge, the etiology of about 40% of birth defects has been recognized to date. Among various birth defects of known etiology, around 36% are caused exclusively by genetic factors, whereas 50–75% results from complex gen-environmental interactions [20]. Around 10% of major types of congenital malformations are attributed to substances of proved teratogenic effects, while 37% to interactions of genetic and environmental factors [21]. Out of 2500 substances that have been recognized as teratogenic agents, only 40 (e.g., carbon monoxide, ozone, lead chromate, lead acetate, lead phosphate, 1,2-dibromo-3-chloropropane, 2-bromopropane) are proven to induce teratogenic effects in humans [22].

### Geographical variations

Geographical variations in prevalence reported by malformation registries may give clues to etiological factors when they represent true differences, as is the observed variation in prevalence rates of neural tube defects in different regions of the world [3]. Geographical variations of birth defects may have several potential contributing factors. Case ascertainment methods, including data collection, sources of information and type of notification of fetal deaths which vary in place and time may contribute to such variations. Demographic and environmental factors may influence the prevalence of congenital anomalies [5].

### Plausible environmental factors in Iraq

Prior to the first Gulf War (1980–1988 Iraq-Iran War) and the second Gulf War (1991 Gulf War) in particular, compared to the west, the Iraqi environment was surely more pristine and environmental challenges were different [23]. The 1991 Gulf War was a prelude to new exposures, including exposure to DU and chemical carcinogens of relevance to the war, and circumstances that could have a teratogenic effect on Iraqi population including sanctions-induced deprivations such as poverty and malnutrition. However, such circumstances without specific chemical or radiological exposures do not lead to increasing rates for certain congenital anomalies [24].

#### Depleted uranium

Much attention has recently focused on worldwide role of DU. The development of DU munitions began around 1959 in the United States and in the early 1960s in the United Kingdom [24]. In the early 1970s, the US army began researching the use of DU metal in Kinetic Energy Penetrators and Tank Armor. DU was ultimately selected due to its availability, price and pyrophoricity [25].

The United States military deployed DU munitions for the first time during the 1991 Gulf War. Depleted Uranium weapons were also used in 1994–1995 war in Bosnia, the 1999 war in Kosovo, the United States 2002 invasion of Afghanistan and the 2003 invasion of Iraq [23]. Apparently the 1991 Gulf War is the only conflict where large DU projectiles were fired from tanks [26].

Military personnel, civilians and the DU munitions producers are being exposed to the DU aerosols that are generated. The detailed pathways, by which environmental DU can be internalized and reach reproductive cells, are not yet fully elucidated. While research on molecular, cellular and animal model level suggests plausibility [23], there is actually no substantial evidence that genetic defects can arise from parental exposure to DU in any circumstances [27]. Inhalation is the most likely route of intake during or following the use of DU munitions in conflict and it may occur as a consequence todecontamination of vehicle from within or near conflict area. Drinking of water or food contaminated with DU is another source. In addition, ingestion of soil by children is also a potentially important pathway. Dermal contact is unimportant pathway because little of DU will pass across the skin into the blood. However, DU could enter the systemic circulation through open wounds or from embedded DU fragments [28].

Preliminary epidemiological studies carried out by community activists in the United States reported hydrocephalic births in a sparsely population rural are downwind of a DU-weapons testing sites [29]. These epidemiological studies raised the possibility of an association between birth defects and exposure to DU. Hydrocephalus is also a component of a congenital malformation syndrome, Goldenhar syndrome, which was reported among infants born to Gulf Wars veterans [30]. Overtime a number of serious health risks and dangerous conditions became linked to DU exposure. These included cancers of different types (leukemias, breast cancer, lymphoma, bone cancer), renal diseases, reactive airway diseases, neurological disabilities, birth defects and perinatal deaths among the neonates of veterans [31].

There is a possibility of genetic damage resulting from exposure to some forms of radiation emitted from particles such as those deposited by DU weapons [32]. Animal studies firmly support the possibility that DU is a teratogen. The fact that DU was detected in the urine of Gulf War veterans 7–8 years after the war is substantial evidence of long-term internal contamination and tissue storage of this substance [31,33].

Basrah is in the region that was heavily exposed to bombardment with DU during the 1991 Gulf War [23]. Two studies from Basrah raised the possibility that DU aerosols were contributing to increase in birth defects by comparison of pre and post 1991 Gulf War data. Both studies reported an apparent increase in the incidence rates from 1995 upwards. The incidence rate of birth defects was 3.04 in 1990 which increased to 7.76 in 1998 and to 13.49 per 1000 births in the period 1999–2000. The incidence of anencephaly raised from 2.5 in 1990 to 3.0 in 1991–94 and 3.6 in 1996–98 [6,7]. Diwaniyah, like Basrah is in the area bombarded with DU munitions during the 1991 Gulf War [23]. Similarly, the Diwaniyah study raises concern about universal maternal exposure to DU [18]. The Diwaniyah study pertains exclusively to NTDs and revealed a high incidence rate of 8.4/1000 births in 2000 in comparison with a rate of 5.4/1000 births during 8- months from 7/98 through 2/99. Although the worker stated that he investigated other possible risk factors for NTDs and they were irrelevant, he did not rule out such risk factors [23].

The low pre 1991 Gulf War baseline incidence of birth defects reported in the Basrah registry studies (3.04 per 1000 births) [6,7] does not necessarily reflect a truly low incidence of birth defects in Iraq at that time, which could be attributed to incomplete registration of birth defects in Iraq. The departments of statistics and registration of hospitals and directorates of health are poor and inefficient. On the other hand, increased awareness and concerns with the problem in post war period might have attributed to over reporting. Such possible over reporting in addition to poor sample representativeness and the generalizability of these findings to the targeted population are significant methodological limitations of the Basrah registry studies. In fact, birth defects were reported to occur at a rate of 10.2 per 1000 births in the prewar period based on reviewing records of deliveries of three maternity hospitals in Baghdad over a period of 18 months from January 1987 through June 1988 [34]. However, this is considered a short period to get a statistically accurate prevalence of such a rare event. The high rates of NTDs reported in Diwaniyah study could also be attributed to economic sanctions imposed in 1990 or similarly to over reporting in postwar period due to increased awareness.

In spite of what has been reported, theoretically Marshall [35] used mathematical modeling to estimate health risks of exposure to DU during the Gulf War and found that the risks of DU-induced birth defect or leukaemia are far too small to result in an observable increase in these health effects among exposed war veterans or Iraqi civilians. In Bosnia and Herzegovina, no significant post-war increase in the prevalence of congenital malformations was detected [36].

Little is known about the types of weapons deployed in Fallujah of Al-Anbar governorate during the first major US assault in April 2004 and during Operation Phanum Fury, the second major assault in November 2004. However, reports began to emerge after 2005 of sudden increase in cancer and leukemia rates [37]. In Al-Ramadi Maternity and Children’s Hospital of Al-Anbar governorate in Iraq, 33 infants were delivered with NTDs (including spina bifida, anencephaly, encephalocele) out of 10016 births between 1^st^ November 2007 and 1^st^ November 2008 giving an incidence of 3.3 per 1000 births [38]. Contamination of parents of children with congenital anomalies and of the environment in Fallujah in western Iraq by Uranium and other elements was investigated by a study group by testing hair samples from 25 fathers and mothers of children diagnosed with congenital anomalies. The study was conducted in response to reports from Fallujah that have drawn attention to increases in congenital birth anomalies and cancer blamed on teratogenic, genetic and genomic stress thought to result from DU contamination following the battles in the town in 2004. Levels of Uranium (for mothers only) and a number of other elements that do not cause congenital diseases were significantly higher than published mean levels in an uncontaminated population in Sweden. The study suggested that exposure to Uranium is either a primary cause or related to the cause of the congenital anomaly and cancer increases [39].

### Polycyclic aromatic hydrocarbons (PAHS)

The issue of disentangling the role of parental exposure to DU from that of exposure to other potential teratogens is serious and complex which needs to be resolved through extensive laboratory and epidemiological researches [23].

In Iraq exposure to other potential teratogens during 1991 Gulf War could not be excluded as the country was highly exposed to chemical carcinogens of relevance to the war. The retreating Iraqi forces set fires to Kuwaiti oil fields, and the generated smoke was carried by prevailing wind over Iraq [40] The environmental damage was huge. The soot would have contained large quantities of polycyclic aromatic hydrocarbons (PAHs). Similarly smoke and soot from burning crude petroleum generated toxic carcinogenic and teratogenic chemical substances like PAHS, dioxins, furan, mercury and sulfur far more than weeks during 2003 invasion operations.

PAHS are potent atmospheric pollutants occur in oil, coal and tar deposits and are produced as byproducts of fuel burning. As a pollutant, they are of concern because some compounds have been identified as carcinogenic, mutagenic and teratogenic. Several studies have reported an association between exposure to PAHs air and adverse birth outcomes including low birth weight and prematurity [41,42]. Studies from Iraq revealed an increasing preterm deliveries and low birth weight following the 1991 Gulf War [43-45]. A report from Kuwait, a major battle- field, showed a significant increase in congenital heart diseases in post 1991 Gulf War [46] which was attributed to environmental damage due to fires set to the oil fields.

#### Dioxins

Chlorinated dibenzo-p-dioxins (CDDs) is a family of released compounds commonly known as chlorinated dioxins. The most toxic compound among CDDs is the 2,3,7,8,TCDD which is odorless [47]. Dioxins are found throughout the world in the environment; the highest levels are found in some soils, sediments, and food especially dairy products, meat, fish and shellfish. Very low levels are found in plants and air [48].

The International Agency for Research on Cancer (IARC), a part of the WHO considered in 1997, 2,3,7,8TCDD a human carcinogen. The health effects of CDDs including birth defects and cancer that observed in animal studies cannot be ruled out in human [49,50]. After Iraq invasion in 2003, the Iraqi Environment Minister said that high levels of dioxins in agricultural lands in south Iraq, in particular, were increasingly thought to be a key factor in a general decline in the health of people living in the poorest parts of the country [51]. Dioxin was the primary component of “Agent Orange”, a herbicide used in the Vietnam War. Paternal exposure to Agent Orange appears to be associated with statistically increased spina bifida [52]. In a total of 22 studies, 13 Vietnamese and 9 non-Vietnamese, the relative risk of birth defects associated with exposure to Agent Orange was 1.95. The degree of association tended also to increase with higher exposure to the agent, rated on intensity and duration of exposure and dioxin concentrations in affected population [53]. Dioxins may share responsibility or responsible for the high reported rates of birth defects in Fallujah after the two major assaults upon this city in 2004 [54].

#### Heavy metals

The possibility of exposure to other specific local risk or causal factors particularly heavy metals needs to be investigated. Animal teratogenesis by a variety of heavy metals and human teratogenesis by at least lead and mercury are long established [55]. In Iraq, several studies described lead exposure and toxicity to lead in Baghdad [56,57] and Al-Anbar governorates [58]. The later study, which examined the blood lead level in 128 children and lead level in 44 samples of water and soil in two areas of Al-Anbar governorate, revealed a high water lead content. However, its association with birth defects was not studied. A study in Glasgow, UK revealed no correlation between exposure to high concentrations of lead in domestic water supply and NTDs [59].

The Fallujah study revealed significant high levels of Ca, Mg, Co, Fe, Mn, V, Zn, Sr, Al, Ba, Bi, Ga, Pb, Hg and Pd in the hair of parents of children with birth defect in comparison with published mean levels in an uncontaminated population in Sweden. Of these only Hg could be considered by the authors as a possible cause of congenital anomaly [39].

#### Chemical weapons

Exposure to chemical agents in the 1980s is another source of exposure. Chemical agents were certainly used during the first Gulf War and in Iraqi Kurdistan against indigenous Iraqi Kurds in 1988; mustard gas being one of these agents [60,61]. On the March 16^th^, 1988 the Iraqi forces attached Halabja with chemical weapons and artillery fire.

Sulfur mustard is an alkylating compound with a potent vesicant property has been used as a chemical weapon in various conflicts during the 20^th^ century. It causes blistering to the skin and mucous membrane with no specific antidote. Mustard gas damage cells through alkylation of DNA and through cytokine-mediated inflammatory response. The long term effects of the exposure to mustard gas are known to include damage to immune system, birth defects and lymphoma [62,63]. An apparent increase in leukemia was reported among war victims [64] and in southern governorates of Iraq [65,66], the major battlefield of first and second Gulf Wars. Investigations have revealed that mustard gas and the nerve gas Sarin, VX, and Tabun were used in Halabja attack [67,68]. However no publications on birth defects among exposed population in Kurdistan region are available.

#### White phosphorus

Incendiary weapons have been issued to US forces in Iraq, apparently mainly to Marine Corps Aviation Wing against Iraqi troops during the 2003 invasion and in Fallujah assault in 2004 [26]. White phosphorus as an incendiary weapon was used in both major assaults on Fallujah [69,70].

Babies born in Fallujah are exhibiting high rates of mortality and birth defects. In September 2009, 170 children were born at Fallujah General Hospital, 24% of whom died within 7 days, 75% of those exhibited deformities including children born with two heads, no head, a single eye in their foreheads or missing limbs. The comparable data for August 2002 recorded 530 births of whom 6 died and only one of whom was deformed [54]. Environmental campaigners believed that either white phosphorus or DU, is a major if not only, cause of birth defects [71].

### Maternal factors

#### Nutritional factors

Maternal nutritional factors have also been identified as an important contributor to neural tube defects [72,73]. Much attention has been focused recently on the role of peri-conceptional folic acid supplementation during pregnancy for the primary prevention of birth defects particularly NTDs [74,75]. Elevated risk of delivering offspring with NTDs following intrauterine famine exposure has been observed [76]. Several studies revealed also an increased risk for delivering infants with NTDs in association with lowered gestational maternal weight gain [77,78]. The UNICEF reported that the availability of calories in the food ration of Iraqi population was equivalent to 1093 calories/person (approximately 40% of daily requirement) for the period 1990–1997 which increased then to 2030 and lastly to 2475 in 2002 [79]. This may have contributed to low maternal weight gain.

#### Maternal age

Maternal age is strongly associated with chromosomal anomalies and the rising proportion of older mothers is likely to contribute to increase in prevalence of anomalies [80,81]. Studies in Iraq reported that late motherhood is increasing in Iraq during sanctions period [82,83].

#### Maternal stress

Recently, it is assumed that the health effects of maternal stress may include increased risk of certain birth defects [84,85]. Stress may exacerbate risk in population with poor nutritional status and meager economic resources [86]. A recent study in Iraq reported exposure of Iraqi pregnant women to a high level of stress during the last two decades [87].

## Conclusions

As no enough data on pre 1991 Gulf War prevalence of birth defects in Iraq are available, the ranges of birth defects reported in the reviewed studies from Iraq most probably do not provide a clear indication of a possible environmental exposure including DU or other teratogenic agents although the country has faced several environmental challenges since 1980.

## Abbreviations

CDD: Chorinated Dibenzo-p-dioxin; DU: Depleted Uranium; NTDs: Neural tube defects; PAHs: Polycyclic aromatic hydrocarbons.

## Competing interests

The authors declare that they have no competing interests.

## Authors' contributions

All authors contributed to the writing, revision and supportive literature reviews in the preparation of this manuscript. All authors read and approved the final manuscript.
